# Oral health and coronary heart disease

**DOI:** 10.1186/s12903-016-0316-7

**Published:** 2016-11-15

**Authors:** Marc J. Mathews, Edward H. Mathews, George E. Mathews

**Affiliations:** CRCED, North-West University, P.O. Box 11207, Silver Lakes, 0054 South Africa

**Keywords:** Coronary heart disease, Periodontal disease, Biomarkers, Integrated model

## Abstract

**Background:**

It is well documented that there is some correlation between poor oral health in the form of periodontal disease and coronary heart disease (CHD). It is unclear whether this correlation is due to a causal relationship or shared underlying disorder such as inflammation. A suitable integrated model of the CHD pathogenetic pathways relevant to periodontal disease may help to elucidate the association. Such a model is currently not available in literature.

**Methods:**

A previously developed integrated model of CHD was used to investigate potential pathogenetic pathways linking periodontal disease to CHD biomarkers.

**Results:**

The integrated model was created to provide insight into possible higher-order biological interactions underlying CHD and periodontal disease. In order to simplify these interactions a novel ‘connection graph’ was developed. It quantitatively illustrates the relationship between periodontal disease and various serological biomarkers of CHD. The pathogenesis of periodontitis shows various possible pathways which could link periodontitis to CHD pathogenesis.

**Conclusion:**

An integrated model of CHD was developed which provides a summary of the potential CHD effects of periodontal disease. Further research must refine and validate the model.

## Background

The largest cause of death globally is Coronary heart disease (CHD) [1]. It is also well documented that poor oral health in the form of periodontal disease is associated with an increased risk for CHD [2–6]. Whether this prevalence is directly linked to a causal effect of periodontitis or the effect of a shared underlying disorder such as inflammation has not been determined [7]. It is however clear that although there is a correlation between periodontal disease and CHD, the extent of this correlation and the usefulness thereof are not yet evident [2–7].

It may thus prove beneficial to quantify and elucidate the underlying pathogenetic effect of periodontal disease on the pathogenesis of CHD. Using a previously described integrated model of CHD [8] we therefore investigated the possible interconnectivity of periodontal disease and the pathogenesis and pathophysiological traits attributed to CHD.

The existing model graphically describes all the hypothetical pathogenetic pathways of CHD [8]. Some of these pathways can then be measured by serum biomarkers to give an indication of the degree of risk such a pathway represents. The model was used in this study to holistically review the interconnections between oral health pathogenesis and the pathogenesis of CHD. The theory is that where these conditions overlap there is the possibility for causal interaction which should be investigated in detail.

The vision behind the model is that it could be validated over time by calibrating it on a patient specific basis. Patients can be compared with themselves over time. Once the sample size of patient specific calibrated models is large enough the model could give new insight on the underlying workings of CHD. However, in the absence of existing studies of our model, using patient specific data, this paper attempts to gain some knowledge by looking at population based data. The data are in terms of risk ratios, the incidence of health factors and typical pathogenesis of CHD. Some interesting insights are revealed about possible interconnections of pathogenesis underlying both CHD and periodontal disease.

## Methods

An integrated model was developed as part of a larger research project [9]. This project was partially described in previous articles dealing with certain subsets of the research [8, 10, 11]. Briefly, an integrated model of CHD and health factors was developed from existing literature. The model was then used to describe the actions of some non-traditional health factors on CHD. This was achieved by using a connection graph which visually shows the relationship between CHD pathogenesis, measurable biomarkers and the health factors.

### Integrated model development

The integrated model of CHD was developed using a systematic review of the literature for CHD pathogenesis, health factors, biomarkers and pharmacotherapeutics. Unless a publication was cited more than 50 times, the resulting dataset included results from 1998 until 2014 as these were deemed to contain the most relevant data. The studies were also selected based on how well they accounted for confounders such as age, sex, socio-economic status and others.

During the systematic literature review PubMed, Science Direct, Ebsco Host, and Google Scholar were searched for publications with “coronary heart disease” or “coronary artery disease” or “cardiovascular disease” or “CHD” as a keyword and combinations with “lifestyle effects”, “relative risk prediction”, “network analysis”, “pathway analysis”, “interconnections”, “systems biology”, “pathogenesis”, “biomarkers”, “conventional biomarkers”, “drugs”, “therapeutics”, pharmacotherapeutics”, “hypercoagulability”, “hypercholesterolaemia”, “hyperglycaemia”, “hyperinsulinaemia”, “inflammation”, and “hypertension” in the title of the study.

Also searched were all major relevant specialty journals in the areas of cardiology, alcohol consumption, nutrition, cigarette smoking, physical exercise, oral health, psychological stress, depression, sleep disorders, endocrinology, psychoneuroendocrinology, systems biology, physiology, periodontology, CHD, the metabolic syndrome and diabetes.

This literature was consulted in detail and the research on the pathogenetic pathways of CHD was extracted therefrom. A total of 118 articles, books and studies were identified as important to the explanation of the pathogenesis of CHD. The information from these studies was thus combined in a graphical manner in order to develop the integrated model of CHD.

The integrated model was populated by considering health factors which were considered as either lifestyle effects or comorbid health disorders. Only health factors which have been associated with statistically significant increases or decreases in CHD risk were included in the model. The health factors considered were only those which are measurable through biomarkers and could conceivably be influenced by medication, prevention or other treatment. Thus, other factors such as socio-economic status were not considered. However, if future models are created for risk prediction more factors such as socio-economic status will have to be considered.

This resulted in nine health factors being considered in the model, namely Alcohol, Food, Exercise, Smoking, Oral health, Stress, Depression, Insomnia and Sleep Apnoea [8, 9].

In brief, the systematic review of literature revealed the pathological effects of various health factors on the pathogenesis of CHD. This information was combined to form a visual representation of the pathogenesis of CHD as it is affected by these health factors. In this study the integrated model was used to describe the possible integrated effects of periodontal disease on the pathogenesis of CHD.

The integrated model of CHD schematically illustrates the complexity of CHD and shows all theoretical pathogenetic pathways between the health factors and CHD. The model has been previously used to describe the effects of high carbohydrate diets on CHD [8], as well as the possible mechanisms through which antidepressants [11] and moderate alcohol consumption [10] may reduce CHD risk.

Furthermore, as the model is already complex, confounders were treated as follows: they were compensated for in the initial data but not included in the model. Therefore, when selecting the data used for the model, only the studies that best accounted for confounders such as age, sex, socio-economic status and others were used.

This encourages focus on a purely measurable biomarker driven model. Such a model, is not yet useful as a practical model for population based risk. It does however give a good indication of the way in which the interconnections between certain measurable factors (biomarkers) and the CHD risk correlate. Potential refining of the model is described in the Discussion section.

### Biomarker identification

In order to allow the model to quantify the functionally measurable aspects of CHD pathogenesis, biomarkers were included in the integrated model [8, 9]. The biomarkers were only those whose measurement has been associated with statistically significant increases or decreases in CHD risk. This resulted in 23 biomarkers being considered in the model, namely triglycerides, low-density lipoprotein (LDL), high-density lipoprotein (HDL), apolipoprotein-B (Apo B), leptin, C-reactive protein (hsCRP), interleukin-6 (IL-6), tumour necrosis factor- α (TNF-α), growth-differentiation factor-15 (GDF-15), osteoprotegerin (OPG), myeloperoxidase (MPO), B-type natriuretic peptide (BNP), homocysteine, fibrinogen, troponins, urinary albumin-to-creatinine ratio (ACR), glycosylated haemoglobin (HbA1c), insulin-like growth factor-1 (IGF-1), adiponectin, cortisol, brain-derived neurotrophic factor (BDNF) and insulin resistance.

### Data selection

When selecting the data for the integrated model, only articles using the following risk measures: relative risk (RR), odds ratio (OR), or hazard ratio (HR) were considered. This study’s intention was to create an overarching model rather than to conduct individual meta-analyses of the individual biomarkers or lifestyle effects. Thus, the risk data used in the model was taken from the most recent and relevant meta-analyses of each biomarker. A single high quality representative study was used where no meta-analysis for CHD risk was available for a specific biomarker or lifestyle effect.

The risk data used in the model was also further selected based on which meta-analysis adjusted for the most confounding variables such as age, sex, socio-economic status and others. This was done in order to ensure that the effects of most of the potential confounders were adjusted for. However, since not all the studies adjusted for the same confounders, this may, have increased the heterogeneity between studies..

For the effects of changes in biomarkers the relative risk (RR) was, where possible, extracted from the most recent meta-analysis conducted on the specific biomarker. If no relevant meta-analysis was available, a suitable high quality study was selected instead. The RR data included in the model was standardised to include only RR data that was given per increase of 1-standard deviation (SD) in the biomarker level. This was done in order to limit errors in comparisons between biomarkers and to prohibit the misrepresentation of risk due to the selection of extreme exposure contrasts [7].

### Pathway characterisation

In this study the integrated model was used to elucidate the potential interactions between periodontal disease and the pathogenesis of CHD. To achieve this, the pathogenesis of CHD in which periodontal disease could play a role is described. A brief summation of the literature reviewed to develop the integrated model is presented as the “pathogenetic effects of periodontal disease” in the results section.

To simplify the importance of these interactions a connection graph was established for the relationship between periodontal disease and CHD. The connection graph is a graphic representation showing how the pathways underlying between CHD and periodontal disease are related to the CHD biomarkers.

The connection graph displays the RR for a 1-SD increase in all the biomarkers. Furthermore, the biomarkers were divided into eight classes, namely vascular function and neurohormonal activity, renal function, necrosis, coagulation, oxidative stress, lipids, metabolic and inflammation markers.

The pathogenesis of periodontal disease, as described by the pathways in the integrated model, was used to determine which of the biomarkers might be affected by periodontal disease. This was indicated on the connection lines between “Oral health” and the biomarkers.

Activation of the pathways can be analysed through measurement of the relevant biomarkers. Thus, to validate the connection graph, data from previous studies investigating changes in serum biomarker levels in patients with periodontal disease was corroborated with the biomarkers indicated in the connection graph.

### Statistical analysis

Statistical analysis of the results was limited to the risk ratios which were extracted from published studies for the various biomarkers of CHD. All risk ratios were extracted to ensure that they were representative of a 1-SD change in the serum biomarker. All risk ratios extracted were converted to RR if given in odds or hazard ratios.

The study makes use of a non-traditional method of graphically displaying the RR indicating a decrease in risk. RR indicating an increasing risk is displayed as per normal. Decreasing RR is transformed by using the inverse of the typically presented RR.

The non-traditional method was used due to the problems of explaining relative risk to an untrained person as a result of the visual scaling of the traditional RR [8]. In this article a conventional RR = 3 is presented as per normal, as a 3-fold increase in risk while a conventional RR = 0.33 is presented as a 3-fold decrease in risk (1/0.33 = 3). This method was used to convert between the RR data extracted from studies and the bar graphs presented in this study.

## Results

### Integrated model of coronary heart disease

The integrated model of CHD which was developed in the previous studies [8–11]. It is presented in Figure One of the article by Mathews et al. [8]. In the integrated model the pathways (pathogenesis of CHD) can be tracked from where a specific health factor influences the relevant tissue, to the end state of CHD. The pathways of the integrated model are thus a visual representation of previously published knowledge on CHD pathogenesis.

**Fig. 1:**
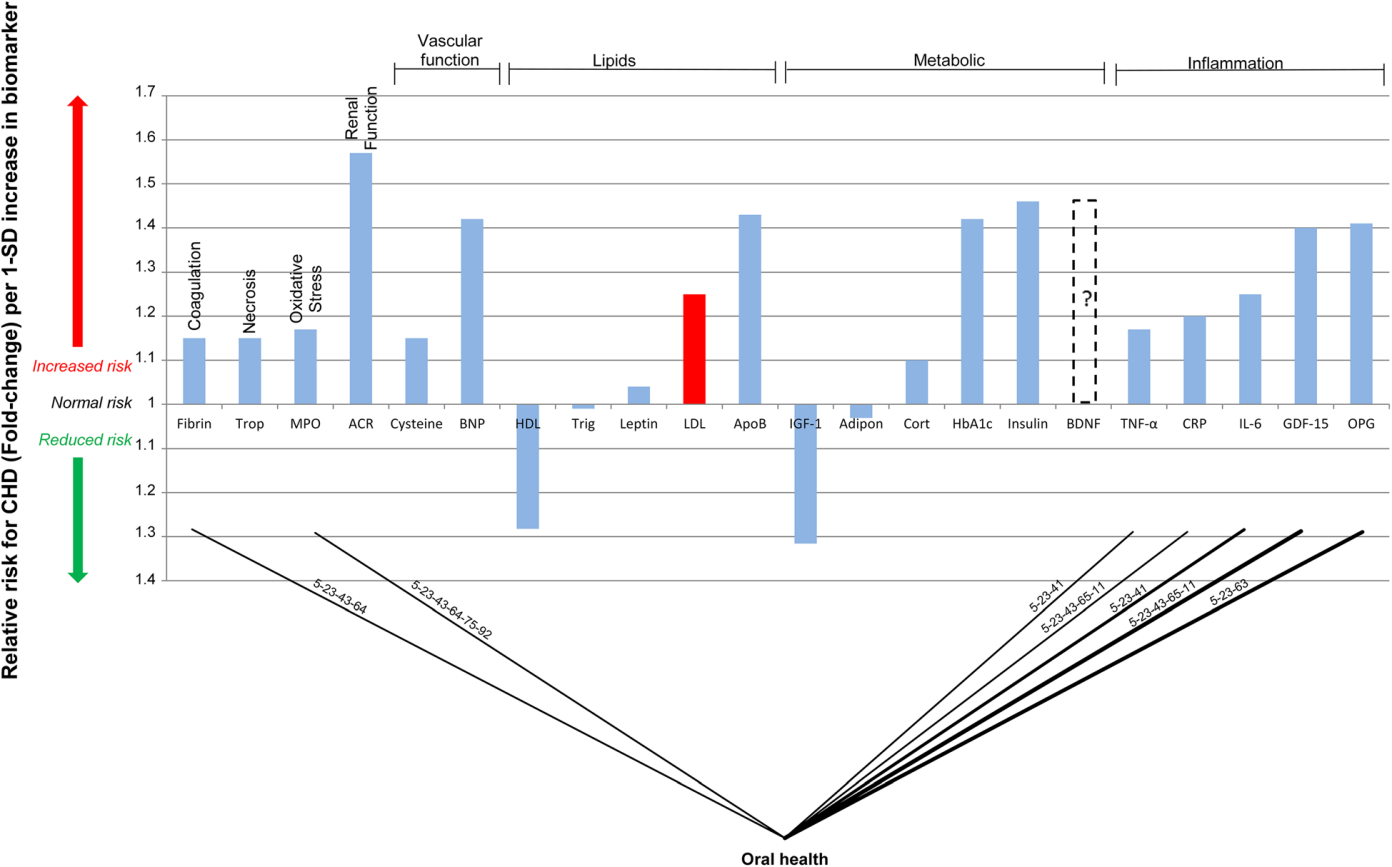
Potential interconnection of relative risk effects of periodontal disease and serological biomarkers for CHD. “ACR” denotes, albumin-to-creatinine ratio; Trop, troponins; Fibrin, fibrinogen; MPO, myeloperoxidase; BNP, B-type natriuretic peptide; Cysteine, Homocysteine; HDL, high-density lipoprotein; LDL, low-density lipoprotein; Trigl, triglycerides; ApoB, Apolipoprotein-B; Adipon, adiponectin; HbA_1c_, glycosylated haemoglobin A1c; Cort, cortisol; IGF-1, insulin-like growth factor-1; BDNF, brain-derived neurotrophic factor; GDF-15, growth-differentiation factor-15; CRP, C-reactive protein; IL-6, interleukin-6; TNF-α, tumour necrosis factor-α; RANKL or OPG, osteoprotegerin

The focus of this paper is on using the integrated model to describe the interconnections of periodontal disease on the pathogenesis of CHD. Thus, a more detailed discussion of Figure One of Mathews et al. [8], relevant to periodontal disease, is given in following sections. This review therefore attempts to quantify the potential CHD effect of periodontal disease by the connection of these to an array of biomarkers which represent increasing or decreasing CHD risk.

### Biomarkers of coronary heart disease

Biomarkers can be used as indicators of an underlying disorder and give an indication of the activation of pathogenetic pathways [12–14]. Furthermore, the prediction of the RR for CHD associated with a biomarker [12–14] is enabled by the measurement of said biomarker’s levels. This can allow for an exploration of the possible effects of periodontal disease on the pathogenesis of CHD by considering the measurement of typically CHD biomarkers in patients with CHD.

The serological biomarkers which were used to simplify the integrated model are given in Table three of Mathews et al. [8]. Figure two of Mathews et al. [8] presents a comparison of the RR for CHD associated with these biomarkers per 1-standard deviation (SD) increase in the biomarker [8, 9].

### Pathogenetic effects of periodontal disease

Figure One of Mathews et al. [8] indicates all possible pathogenetic pathways between the considered lifestyle effects and CHD. In the present paper we only appraise the possible CHD effects of periodontal disease. These are noted in Table 1 and will be described in detail.

**Table 1:** Putative effects of periodontal disease and salient CHD pathogenetic pathways

Pathways, and pathway numbers corresponding to those in Figure One of Mathews et al. [8]	Refs.
a. 5-23-↑P. gingivalis-43-↑periodontitis-64-↑platelet factors-73-↑hypercoagulability	a. [2, 4, 5, 64, 65]
b. 5-23-↑P. gingivalis-43-↑periodontitis-65-↑oxLDL-51-↑hypercholesterolaemia	b. [2, 4, 5, 64, 65]
c. 5-23-↑P. gingivalis-43-↑periodontitis-62-↑ROS-85-↑inflammatory state	c. [2, 4, 5, 64, 65]
d. 5-23-↑P. gingivalis-43-↑periodontitis-41-↑TNFα/IL6-↑inflammatory state	d. [4, 5, 64, 65]
e. 5-23-↑P. gingivalis-43-↑periodontitis-41-↑TNFα/IL6-↑inflammatory state-60-↑insulin resistance	e. [2, 64, 66, 67]
f. 5-23-↑P. gingivalis-43-↑periodontitis-41-↑TNFα/IL6-↑inflammatory state-60-↑insulin resistance-↑vasodilation	f. [68]

↑ denotes up regulation/increase, ↓ denotes down regulation/decrease, x-y-z indicates pathway connecting x to y to z. *FFA* free fatty acids, *IGF 1* insulin-like growth factor-1, *IL6* interleukin-6, *LDL* low-density lipoprotein, *MAPK* mitogen-activated protein (MAP) kinase, *MCP 1* monocyte chemoattractant protein-1, *NO* nitric oxide, *oxLDL* oxidised LDL, *P. gingivalis* Porphyromonas gingivalis, *PI3K* phosphatidylinositol 3-kinase, *PI3K:MAPK* ratio of PI3K to MAPK, *ROS* reactive oxygen species, *SMC* smooth muscle cell, *TNFα* tumour necrosis factor-α, *VCAM 1* vascular cell adhesion molecule-1

Many of the potential pathogenetic effects of periodontal disease on CHD have been postulated to be due to the entry of bacteria or bacterial products into the blood stream [5]. A common periodontitis associated bacteria, *Porphyromonas gingivalis* (*P.gingivalis*), has been found to invade endothelial cells [15] as well as atheromatous tissues [16, 17]. This is one of the pathogenetic links between periodontal disease and CHD as shown by *pathway: 5-23- P.gingivalis* in the integrated model in Figure One of Mathews et al. [8]. In order to consider the overall effect of periodontal disease on CHD we will therefore consider all pathogenetic links between the two.

One of the possible links between *P.gingivalis* and CHD, supported by in vitro testing, is through increased platelet activity via a TLR2-dependent mechanism [18]. *Pathway 5-23-P. gingivalis-43-periodontitis-64-platelet factors-73-hypercoagulability* in Figure One of Mathews et al. [8] shows how increased platelet activity from periodontal disease can lead to an increased possibility for hypercoagulability, a hallmark of CHD.

Pathway *5-23-P. gingivalis-43-periodontitis-65-oxLDL-51-hypercholesterolaemia* in Figure One of Mathews et al. [8] shows how periodontal disease can have an effect on oxidised LDL cholesterol (oxLDL) due to the increased reactive oxygen species (ROS) associated with periodontal disease [19]. Increased ROS up regulates the oxidation of LDL cholesterol to form oxidised LDL [2].

Increased ROS may also play a crucial role in the link between periodontal disease and systemic inflammation. Pathway *5-23-P. gingivalis-43-periodontitis-62-ROS-85-inflammatory* in Figure One of Mathews et al. [8] shows how increased ROS can activate nuclear factor-κB (NF-κB) and consequent production of growth factors and pro-inflammatory cytokines [2] leading to systemic inflammation.

It is also possible that chronic systemic inflammation can further be up regulated by *P.gingivalis*, by causing increased elevations in C-reactive protein [20], fibrinogen [21], tumour necrosis factor-α (TNF- α) and intereukin-6 (IL-6) [4, 5] serum levels. These actions are shown in Figure One of Mathews et al. [8] by *5-23-P. gingivalis-periodontitis-41-TNFα/IL6-inflammatory state.*

Increased and systemic inflammation could link periodontal disease and a pro-atherogenetic state of insulin resistance. Pathway *5-23-P. gingivalis-43-periodontitis-41-TNFα/IL6-inflammatory state-60-insulin resistance* in Figure One of Mathews et al. [8] shows how the release of pro-inflammatory cytokines such as TNF-α, IL-6 and IL-1 from inflamed periodontal tissue could induce insulin resistance [22–24].

Pathway *5-23-P. gingivalis-43-periodontitis-41-TNFα/IL6-inflammatory state-60-insulin resistance-vasodilation* shows how periodontal disease could be linked to changes in vasodilation. Increased insulin resistance as a result of inflammation could affect vasodilation by impairing the vasodilation effect of insulin [25, 26].

It is thus evident from the integrated model that there are significant potential share underlying pathogenetic links between periodontal disease and CHD. These are largely in the form of increased inflammation and potential changes in hypercoagulability and insulin resistance. The rest of this paper will attempt to quantify the importance of the connections and links identified above by considering the changes in biological markers.

### Effects of periodontal disease

In the previous section we elucidated the pathogenetic pathways underlying to CHD which are potentially activated by periodontal disease. Now we will link these pathways to measurable CHD biomarkers. It is possible that a pathogenetic pathway may be shared by both CHD and periodontal disease. However periodontal disease may not have a measured effect on CHD risk biomarkers for this pathway. This may indicate that the pathogenetic pathway is not influenced by periodontal disease in such a way which would increase CHD risk.

In an attempt to validate the theoretical pathways we considered existing literature which has shown measured differences in the serological biomarkers of CHD risk in patients with periodontal disease. Using this to describe the potential connection between periodontal disease and the serological biomarkers of CHD enables the simplification of the integrated model into a ‘connection graph’. The connection graph in Fig. 1 shows all the potential connections between periodontal disease and the measurable serological biomarkers of CHD. The RR values linked with the relevant biomarkers were given in Table three of Mathews et al. [8].

The pathways from the integrated model (Figure One of Mathews et al. [8]) which are regulated by periodontal disease and described in Table 1 are thus shown on the connecting lines in Fig. 1. Each pathway suggests the manner in which the CHD biomarker is likely affected by periodontal disease. Previously published research on changes in serum biomarkers in patients with periodontal disease was used in an attempt to validate the connections between periodontal disease and the CHD biomarkers.

The explanation of the pathogenetic pathways in Table 1 would suggest that there should be a strong connection between periodontal disease and the markers of systemic inflammation. It is also evident from the connection graph of periodontal disease (Fig. 1), that there are a significant number of connections to the inflammatory biomarkers due to periodontal disease.

Numerous studies have noted increased serum levels of CRP in patients with periodontal disease compared to those without [20, 27–29]. One study [27] found elevations of CRP levels in the order of 30% when compared to patients without periodontal disease. Increased CRP levels are significantly associated with an increase in CHD risk (See Table three of Mathews et al. [8]).

Additionally, there is production of proinflammatory cytokines such as IL-6 and TNF-α in inflamed periodontal tissue [30, 31], which can further antagonise a systemic inflammatory response [32, 33]. Increased levels of IL-6 and TNF-α are both linked to increased risks for CHD (See Table three of Mathews et al. [8]).

In severe cases of inflammatory response, where inflammation has spread to the alveolar bone, proinflammatory cytokines can induce bone loss by increasing the expression of RANKL [34] and thus influence the levels of Osteoprotegerin (OPG) [35]. Decreased OPG levels, as a surrogate of RANKL levels, have been found to be significantly associated with increased CHD risk (see Table three of Mathews et al. [8]) [36]. It is thus possible that RANKL or OPG serum levels may serve as an indication of the severity of inflammation present due to periodontitis.

The connection graph in Fig. 1 shows that a strong connection between periodontal disease and inflammation is postulated and the abovementioned research confirms this. This indicates that it may be possible that a systemic inflammatory response to periodontal disease can antagonise CHD and CHD risk [12, 37].

Insulin resistance is postulated to be affected indirectly by periodontal disease through the actions of inflammatory cytokines [22–24]. Increased insulin resistance has indeed been found to be associated with the severity of periodontal infection [24]. Insulin resistance is however known to increase CHD risk [38] and Fig. 1 shows the connection between periodontal disease and insulin resistance with reference to the pathway from the integrated model of CHD (Figure One of Mathews et al. [8]).

It is known that chronic periodontal disease increases reactive oxygen species (ROS) generation which in turn depletes plasma antioxidants [2] and causes an oxidative stress situation [39]. This has been noted by increases in myeloperoxidase (MPO) in patients with chronic periodontal disease [40, 41] and is shown by the connection in Fig. 1. Oxidative stress induced by excess ROS could increases CHD risk due to the increased oxidation of LDL by ROS into oxidised LDL [42].

Oxidised LDL has been implicated in the pathogenesis of atherosclerotic plaques by facilitating cholesterol uptake by macrophages and the formation of foam cells in the endothelium [42, 43]. Therefore, MPO as an indicator of oxidative stress is attributed with a significant increased risk for CHD (See Table three of Mathews et al. [8]) [44].

Increases in serum fibrinogen levels have been found in patients with chronic periodontitis [45]. It is known that elevated serum levels of fibrinogen are implicated in poor CHD prognosis and increased CHD risk [46]. Increased fibrinogen caused by periodontal infection may cause an underlying increase in CHD risk by increasing the possibility for hypercoagulability as shown in Fig. 1.

## Discussion

The use of an integrated systematic approach to better understand CHD elucidates the potential pathways which could lead to the hallmarks of CHD and a CHD event. It is evident that the pathological effects of periodontal disease are in some cases similar to or antagonise the pathogenesis of CHD.

It is apparent from Fig. 1 that there are several possible links between periodontal disease and CHD. Connections are evident between periodontal disease and CHD biomarkers through the hallmarks of hypercoagulability, and an inflammatory state.

From the connection graph (Fig. 1) it would appear that these connections may be relevant to CHD risk. However, what is not apparent is whether these connections are relevant to increased risk for CHD and if the relationship is causal. It is possible that these links may be mediated by direct pathological effects of periodontal bacteria or through the effects of underlying disorders such as inflammation.

These relationships should therefore be tested on a population basis in suitable trials. For instance, a study which determines the relationship between periodontal disease, CHD and serum biomarker levels would be needed to more clearly determine this relationship. Such a study would need to recruit a sample population having periodontal disease and note changes in the relevant biomarker levels with varying levels of periodontal disease. Furthermore, the study population must be large enough to limit confounding and the study period long enough for CHD incidents to occur. Unfortunately such studies are outside the scope of our research group.

Smaller studies could identify the effect of the successful treatment of periodontal disease on inflammatory markers, insulin sensitivity and markers of coagulation and oxidative stress (Fig. 1). While these will not substantiate a causal relationship between CHD and oral health they will show the relationship between the underlying pathogenetic pathways and periodontal disease with reference to known measurable CHD risk biomarkers.

Furthermore, the model presented may elucidate important CHD biomarkers which should be measured in patients with periodontal disease to more adequately screen for CHD risk, namely HOMA, TNF-α, CRP, IL-6, GDF-15, OPG, MPO and fibrinogen. This could lead to better treatment of patients at risk of CHD. In addition other risk confounders such as age, sex and socioeconomic status would need to be considered.

These other risk considerations are important for further development of the integrated model of CHD. For instance, it is known that some of the health factors we have included in the model are affected by confounders such as age, sex and socioeconomic status [47–49]. In particular, socioeconomic status is a substantial confounder to using the model on a population basis for risk prediction. It has the potential to present a significant confounding effect on all the health factors [49–53].

These further risk considerations should be included if the model is used for risk prediction on a population scale. It is important because these further considerations such as socioeconomic status are mediators of the health factors in the model and thus may present a top down approach to intervention.

Currently the integrated model is based purely on available measurable biomarker data and as such it is not meant as a practical model. Thus, the current model cannot be used for practical risk prediction of large populations until factors such as age, sex, socio-economic status and others are taken into account [54]. However, it is theorised that the model could be validated on a patient specific basis by comparing the patient specific biomarkers and health factors with themselves over time. Such an approach would circumvent the problem of confounders such as such as age, sex, socio-economic status by using the patient as their own ‘control’.

A study could be conducted which attempts to validate the theoretical pathways by comparing a patient with themselves at different times where by different health factors or severity of health factors may be applicable. Such a study will require a smaller sample size but the results of the study would not likely be applicable to a greater population. However, it could validate the theoretical model to such a degree that large trials may prove worthwhile.

As such the current model is based entirely on a ‘theoretical person’ devoid of external factors. This theoretical model is built on data taken from external sources with an emphasis on having removed the most confounders in order to focus just on the underlying connections. The current integrated model is therefore more geared towards showing that these interconnections exist between health factors and CHD in a holistic sense. These should be studied further in preparation for the creation of a practical tool.

The intention of the model was to determine the likely method of action for a possible causal relationship between periodontal disease and CHD. In this regard the model itself shows why a causal relationship has not yet been demonstrated due to the interconnected nature of the disorders. The model shows how many variables mediate or confound the relationship. Each of these variables will have to be accounted for in studies of suitable size and design.

However, even with these shortcomings, numerous studies have alluded to the fact that periodontal disease is associated with a statistically significant increase in CHD risk [2–6]. The effect of periodontal disease on CHD biomarkers as shown in Fig. 1 indicates some of the effects which could serve to explain some of the risk for CHD. It is known that when increases in these biomarkers are considered in isolation they are associated with increased risk for CHD [36, 38, 46, 55, 56].

The importance of these relationships are however not immediately clear. An example may provide some context to their importance. An interesting comparison to make is between the relative risk for CHD associated with elevated LDL-cholesterol and the relative risk for CHD associated with periodontal disease. This in combination with the percentage of the population at risk of high cholesterol or periodontal disease should make the comparison interesting.

Previous studies have shown that the increased CHD risk attributed to periodontal disease is a relative risk of 1.34 (confidence interval 1.27 to 1.42) [6]. Other studies have shown that the risk for typical elevations (1-SD above the mean) in LDL-cholesterol is 1.25 (confidence interval 1.18 to 1.33) [57]. The study mean for LDL-cholesterol was 115.5 mg/dl and 1-SD was 35.0 mg/dl so this relative risk correlates to a LDL-cholesterol level of 150.5 mg/dl.

Considering that moderate periodontitis is present in 30.0% of the adult population of the United States [58] there is a significant 34% greater risk for them than those without periodontal disease. Knowing that LDL-cholesterol is widely regarded as the first indication of CHD risk [57, 59, 60] it would stand to reason that many more people are at risk due to high levels of LDL-cholesterol. If we look at the research 31.7% of the United States population is at risk of high LDL-cholesterol (above 130 mg/dl) [61]. This equates to an increased risk of less than 25% (1.25 for LDL greater than 150 mg/dl).

The CHD risk associated with LDL-cholesterol serum levels have resulted in substantial initiatives aimed at reducing LDL-cholesterol to reduce CHD risk [59, 62]. Considering that the potential CHD risk for periodontal disease may be even greater would it not be appropriate to engage in similar research undertakings to investigate if periodontal disease should be positioned as an important risk factor for CHD?

Various studies have investigated the effect of periodontal treatment on an array of CHD biomarkers. It was found that periodontal treatment significantly improved biomarkers of CHD [63]. However, causality between periodontal disease and CHD has not been suitably tested. If a causal relationship between periodontal disease and CHD could be substantiated it would elevate periodontal disease to an important CHD risk factor.

## Conclusion

Although there is evidence that periodontal disease is associated with a higher risk of CHD, all the possible effects on CHD pathogenesis are not available in a detailed integrated model. Such a model should help provide further insight. A high level conceptual model was thus developed which shows possible links of periodontal disease with the pathogenesis, hallmarks and biomarkers of CHD.

It was shown that periodontal disease has significant physiological effects which are similar to or antagonise the pathogenesis of CHD. Important effects are increased inflammation, increased coagulation and insulin resistance. These pathological actions are backed up by the changes which have been noted through measured biomarkers.

These pathways and actions may explain why a significant risk for CHD has been observed in patients with periodontal disease. This is specifically important considering the large percentage of the United States population which have some form of periodontal disease. However, more research is needed to conclusively substantiate a causal link between periodontal disease and CHD.

## Abbreviations

ACE: Angiotensin-converting-enzyme
ACR: Albumin-to-creatinine ratio
Apo B: Apolipoprotein-B
BDNF: Brain-derived neurotrophic factor
BNP: B-type natriuretic peptide
CHD: Coronary heart disease
COX: Cyclooxygenase
CRP: C-reactive protein
D-dimer: Fibrin degradation product D
FFA: Free fatty acids
GCF: Gingival crevicular fluid
GDF: Growth-differentiation factor
HbA_1c_: Glycosylated haemoglobin A_1c_
HDL: High-density lipoprotein
Hs: Homocysteine
ICAM: Intracellular adhesion molecule
IGF-1: Insulin-like growth factor-1
IL: Interleukin
LDL: Low-density lipoprotein
MAPK: Mitogen-activated protein kinase
MCP: Monocyte chemoattractant protein
MIF: Macrophage migration inhibitory factor
MMP: Matrix metalloproteinase
MPO: Myeloperoxidase
NF-κB: Nuclear factor-κB
NO: Nitric oxide
NO-NSAIDs: NO-non-steroidal anti-inflammatory drug
OPG: Osteoprotegerin
oxLDL: Oxidised LDL cholesterol
P. gingivalis: Porphyromonas gingivalis
PAI: Plasminogen activator inhibitor
PDGF: Platelet-derived growth factor
PI3K: Phosphatidylinositol 3-kinase
RANKL: Receptor activator of nuclear factor kappa-beta ligand
ROS: Reactive oxygen species
RR: Relative Risk
SCD-40: Recombinant human sCD40 ligand
SD: Standard deviation
SMC: Smooth muscle cell
SSRI: Serotonin reuptake inhibitors
TF: Tissue factor
TMAO: An oxidation product of trimethylamine
TNF- α: Tumour necrosis factor-α
VCAM: Vascular cell adhesion molecule
vWF: von Willebrand factor
